# A Network of Splice Isoforms for the Mouse

**DOI:** 10.1038/srep24507

**Published:** 2016-04-15

**Authors:** Hong-Dong Li, Rajasree Menon, Ridvan Eksi, Aysam Guerler, Yang Zhang, Gilbert S. Omenn, Yuanfang Guan

**Affiliations:** 1 Department of Computational Medicine and Bioinformatics, University of Michigan, Ann Arbor, Michigan, United States; 2 Institute for Systems Biology, Seattle, Washington, United States; 3 Department of Internal Medicine, University of Michigan, Ann Arbor, Michigan, United States; 4 Department of Electrical Engineering and Computer Science, Ann Arbor, Michigan, United States

## Abstract

The laboratory mouse is the primary mammalian species used for studying alternative splicing events. Recent studies have generated computational models to predict functions for splice isoforms in the mouse. However, the functional relationship network, describing the probability of splice isoforms participating in the same biological process or pathway, has not yet been studied in the mouse. Here we describe a rich genome-wide resource of mouse networks at the isoform level, which was generated using a unique framework that was originally developed to infer isoform functions. This network was built through integrating heterogeneous genomic and protein data, including RNA-seq, exon array, protein docking and pseudo-amino acid composition. Through simulation and cross-validation studies, we demonstrated the accuracy of the algorithm in predicting isoform-level functional relationships. We showed that this network enables the users to reveal functional differences of the isoforms of the same gene, as illustrated by literature evidence with *Anxa6* (annexin a6) as an example. We expect this work will become a useful resource for the mouse genetics community to understand gene functions. The network is publicly available at: http://guanlab.ccmb.med.umich.edu/isoformnetwork.

Genes fulfill their functions by interacting with each other through complex biological networks of proteins. Gene functions or networks can be studied both experimentally and computationally. A key approach to systematically model gene interactions is to establish functional relationship networks^1^,^2^,^3^,^4^,^5^, which present the probability of two proteins working in the same biological process. Our previous work has generated gene-level functional relationship networks for the mouse^3^,^6^,^7^, which have been used by the mouse genetics community to guide the discovery of disease-associated genes. For example, the recent discovery of *Hydin* (axonemal central pair apparatus protein) as a novel thermal pain gene^8^ was guided by our networks developed for the mouse. Multiple methods have been proposed to predict gene/protein functions. For example, a computational approach predicts protein functions through integrating domain features, domain interaction and domain co-existence information^9^.This approach was shown to improve both prediction accuracies and annotation reliability^9^. A method to mine protein surface pocket similarity networks predicted Gene Ontology (GO) functions with high accuracies^10^. These efforts have been focused on gene-level integration. As for isoform-level studies, an interesting method called SpliceNet was proposed to identify isoform-specific co-expression networks^11^. Applying this method to normal and non-small cell cancer samples, network rewiring was identified exemplified by the networks centered around BCL-X and EGFR^11^. In other recent work, an isoform-isoform interaction database was developed as a resource for studying protein-protein interactions (PPI) at the isoform level through integrating RNA-seq, domain and PPI data^12^. Through integrating many RNA-seq datasets, a computational approach was proposed to study splicing modules and to predict isoform functions^13^. Using the multiple-instance based label propagation approach, functional annotation of human isoforms was conducted in a genome-wide manner^14^. These studies represent important advancement towards isoform-level understanding of gene functions. Functional networks are an important approach for understanding gene functions, but remain mostly unexplored. Such functional networks providing higher resolution, at the splice isoform level are essential to understand disease mechanisms^15^,^16^.

The pipeline developed for gene-level networks cannot be readily extended to establishing networks at the isoform level due to two major obstacles. First, most of the traditional functional genomic data, such as most microarray expression and physical interactions, are routinely recorded or analyzed at the gene level, and thus do not directly provide isoform-level features. Fortunately, recent development of computational approaches and experimental technologies has provided multiple types of genomic data sources at the isoform level, including RNA-seq^17^,^18^,^19^,^20^. We also included the computationally predicted isoform-isoform docking scores^21^ previously developed by co-authors of this work, which achieved the top performance in the CASP (Critical Assessment of protein Structure Prediction) benchmark study. The availability of these data provided a solution to the first obstacle.

The second challenge facing isoform-level network modeling remains: we do not have a large set of functionally related isoform pairs to serve as the gold standard for evaluating and integrating large-scale genomic data. Of interest, recent studies have analyzed protein-protein interactions at the isoform level^22^,^23^, providing high-resolution protein interaction data. But the resulting data size is not sufficient to be used as a gold standard to build a genome-wide isoform network. Both biological functions^24^ and pathways^25^,^26^,^27^,^28^,^29^ are conventionally documented at the gene level rather than at the isoform level, preventing any classical method developed for building gene-level networks from being directly applied to splice isoforms.

In this paper, we used a Bayesian network-based multiple-instance learning (MIL) algorithm to solve this problem. MIL formulates a gene pair as a bag of multiple isoform pairs of potentially different probabilities to be functionally related, in analogy to a gene considered as a bag of isoforms of different functions^15^,^30^,^31^,^32^. Our work is focused on constructing isoform-level functional relationship networks (FRN) for the mouse, which significantly differs from previous studies^11^,^12^ in terms of both computational approaches and research content. The SpliceNet focused on building isoform-level coexpression networks using large dimensional trace and identifying differential networks between control and cancer samples^11^. The IIIDB work^12^ used a gold standard of physical interaction data from the IntAct database^33^, and built logistic regression models to predict human isoform-level physical interaction networks. Using the proposed approach, we have built an isoform-level network for the mouse through heterogeneous data integration and validated it through both cross-validation and literature evidence^31^,^34^.

## Methods

### A multiple-instance learning algorithm for predicting isoform networks

**Figure 1:**
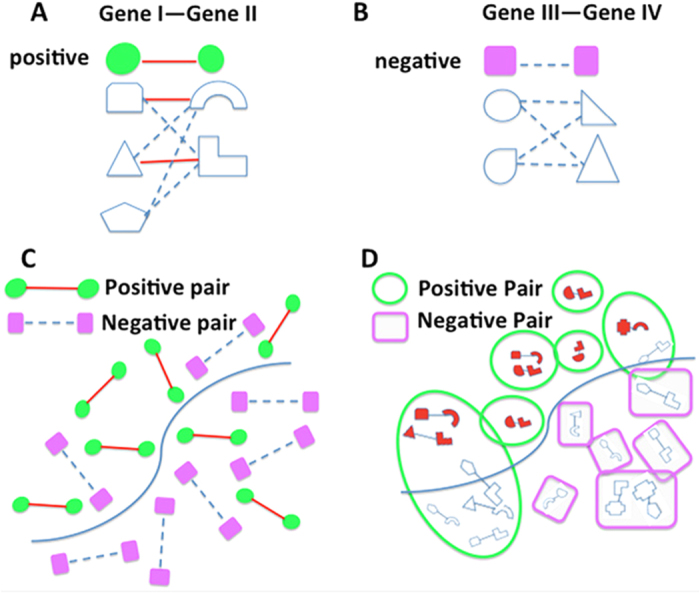
Formulating the isoform-level network prediction into a multiple instance learning (MIL) problem. (**A**) Illustration of a functionally related gene pair (a positive bag), gene I with 3 isoforms and gene II with 2 isoforms. Among these, two isoform pairs are functionally related (solid red line), while the other four isoform pairs have no functional relationship (dashed light blue line). (**B)** Illustration of a functionally unrelated gene pair (a negative bag), gene III with 2 isoforms and gene IV with 2 isoforms. None of the isoform pairs between gene III and IV is functionally related. (**C)** In the traditional gene-level network prediction, a classification model is built to distinguish positive pairs from negative pairs. (**D)** In the isoform-level network prediction using MIL, gene pairs are considered as ‘bags’, each containing one or several isoform pairs, called ‘instances’. A positive bag must have at least one of its instances (isoform pairs) being functionally related, which are called ‘witnesses’ (pairs in red). All instances (isoform pairs) in a negative bag must be functionally unrelated. MIL gives predictions at both bag and instance level.

The key challenge facing isoform-level network modeling is the lack of ground-truth functionally related isoform pairs. To solve this problem, following our recent work in predicting isoform functions^15^,^30^, we assume that: (i) of a functionally related gene pair (a positive bag), at least one of its isoform pairs (the instances) must be functionally related (Fig. 1A); (ii) for an unrelated gene pair (a negative bag), none of its isoform pairs can be functionally related (Fig. 1B). Then, the aim is to identify the truly functionally related isoform pairs (“witnesses”) of the positive bags (Fig. 1). Under these two assumptions, the isoform network prediction is formulated as a MIL problem.

MIL can embed any base learner, such as support vector machines (SVM)^35^,^36^,^37^ and Bayesian network classifiers^7^,^38^. Because genomic datasets are characterized by large scale, heterogeneity, and missing data, the state-of-the-art naïve Bayesian network was chosen as the base learner of MIL since it has been proven promising for data integration in the functional genomics field^39^,^40^,^41^,^42^,^43^. An extended version of naïve Bayesian networks is to incorporate conditional dependencies among features into the network model, which was shown to be a powerful approach in predicting eukaryotic transcriptional cooperativity, a type of functional networks between transcriptional factors^44^. One limitation of previously developed MIL algorithms is that they treat all isoform pairs or randomly choose one isoform pair in a positive bag as the witnesses in the initial iteration, which is very likely to introduce false positives^30^,^35^. To overcome this issue, we developed a single-instance bag MIL (SIB-MIL) algorithm which achieved the best performance in this context compared to the previous methods (Supplementary Figure S1). The algorithm is detailed in the following.

Without loss of generality, the *i*th gene pair containing *m* isoform pairs is denoted by 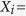 {x_*i*1_, x_*i*2_⋯x*_im_*} with **x**_*ij,*_ *j* = 1, 2 … *m* denoting the *j*th isoform pair of the *i*th gene pair. We assign the class label of the *i*th gene pair, denoted as *y*_*i*_, based on the hypotheses that for a positive gene pair, at least one of its isoform pairs is functionally related; if a gene pair is negative, none of its isoform pairs should be functionally related, which can be mathematically expressed as follows:


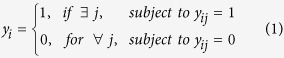


where *y*_*i*_ indicates the label of the *j*th isoform pairs of the *i*th gene pair. We refer to those positive instances (functionally related isoform pairs) of a positive bag (positive gene pair) as the witnesses. The MIL algorithm consists of three steps:

(1) Initialization: because there is no existing isoform-level gold standard, a set of isoform pairs in positive gene pair bags needs to be selected as witnesses to build an initial model. Motivated by the fact that the isoform pair in single-instance positive gene pair bags must be positive, all the instances in such bags are labeled as Class 1 (functionally related). All instances in negative bags are labeled as Class 0 (functionally unrelated). In doing so, unlike the algorithms in^35^, no false positives will be introduced in this step.

(2) The loop:

   (2.1) Model building: using the current witness set and the negative isoform pairs, we build a Bayesian network classifier that will be used to re-assign a probability score to all instances in the original training set.

   (2.2) Witness updating: For each positive bag, reselect the instance with the maximum probability score as the “witness” and label it as Class 1. For each negative bag, only the highest scored instance was chosen and labeled as Class 0; the reason is that a classifier is expected to perform well if it can correctly classify the most difficult examples.

(3) Stop criteria and final predictions: The iteration is stopped when cross-validation performance does not change any more. The final classifier, built at the instance (isoform pair) level, will be used to predict the isoform network. Each isoform pair will be assigned with a probability to be functionally related. At the gene-pair bag level, the score of each bag is defined as the maximum of all scores of its instances.

We used a Bayesian network classifier, as previously described in the work^7^,^41^,^45^,^46^ as the base learner. Assuming that each isoform pair is characterized by an *n*-dimensional feature vector (*E*_1_, *E*_2_, … *E*_n_), the probability with which an isoform pair belongs to the positive class can be calculated using the Bayesian formula^7^,^47^:


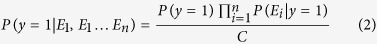


where *P*(*y* = 1) is the prior probability for an isoform pair to be positive, *P*(*E*_*i*_|*y* = *1*), i = 1, 2, …, n, is the probability of the *i*th feature value, conditioned that the isoform pair is functionally related, and *C* is a constant normalization factor.

### Isoform-level genomic data processing and gold standard construction

We have processed 65 heterogeneous datasets, including RNA-seq data, exon array, pseudo-amino acid composition and isoform-docking data. For the isoform docking data, the docking score between a protein pair is used as the feature data. For the other three types of datasets, we calculated the correlation between each isoform pair and used it as the feature input. Details for processing these four types of data into pairwise features are described in Supplementary Text S1. The data integrated in this work are listed in Supplementary Table S1. These four types of feature datasets together provide a largely comprehensive characterization of isoform pairs.

In our study, a gene is assumed to carry a function if it is annotated to a biological process or a pathway. Two genes are assumed to have a functional relationship if they are co-annotated to the same biological process or pathway. We constructed a gene-level gold standard of functionally related pairs using the Gene Ontology (GO)^24^, KEGG^25^, and BioCyc^27^ databases; 675, 124 positive gene pairs were obtained in total (Supplementary Text S1). Functionally related and unrelated gene pairs are called positives and negatives, respectively. Consistent with previous work in this field^7^,^38^, negative gene pairs are generated randomly.

## Results

### Simulation study shows accurate prediction of functional relationships at the isoform level

**Figure 2:**
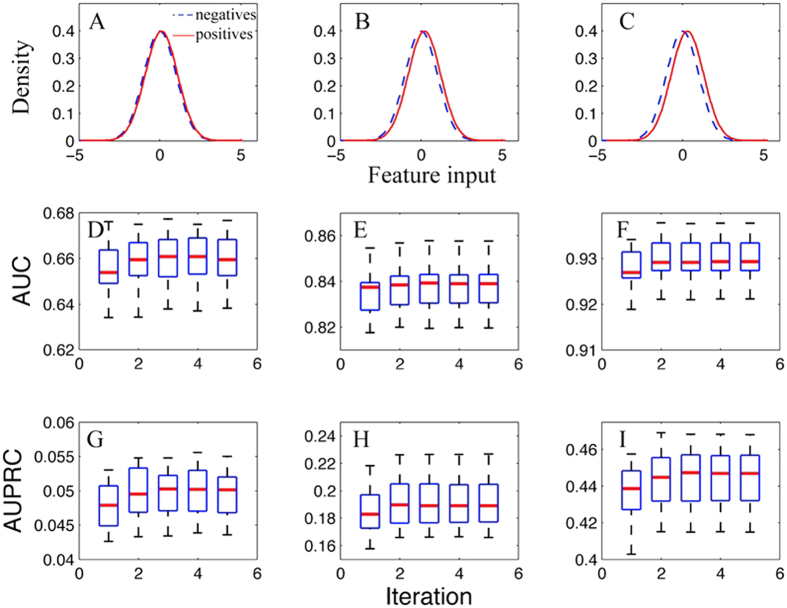
Isoform pair level predictive performances of multiple-instance learning on the simulated data at different values of mean difference of feature inputs between functional related (positives) and unrelated (negatives) isoform pairs. The multi-isoform ratio was fixed at 0.3. For both the positives and the negatives, the distribution of feature input was simulated with a normal distribution with standard deviation of 1. (**A**–**C)** shows the distribution of the feature inputs for the positives and the negatives, with mean difference = 0.1, 0.2 and 0.3 respectively. AUC and AUPRC are shown in (**D**–**I**).

We first tested the performance of this algorithm using simulated data under different scenarios (Supplementary Text 2). Two parameters were tested in the simulation study: (1) the discriminativeness of the input data, measured by the mean difference (MD, see Fig. 2A–C),) of the values between the population of functionally related isoform pairs and the population of functionally unrelated isoform pairs and (2) the multi-isoform gene ratio (MGR), defined as the ratio of multi-isoform genes to the total number of genes (Supplementary Text 2). Though studies have shown that around 95% of multi-exon genes have isoforms^48^, we tested MGR values of 0.2, 0.3 and 0.5 for the reason that the NCBI RefSeq gene build used in this study (version 37.2) contains only high quality isoforms and therefore has many fewer annotated transcripts compared with the alternative. Ensembl gene model. The GTF file downloaded from the cufflinks website has only 3505 multi-isoform genes and 22287 single isoform genes, giving a MGR only 13.5%. For each simulation, we randomly partitioned gene pairs into disjoint training and test set, respectively. We repeated the partitioning 20 times and thus tested this method on 20 randomly generated test sets. For each partition, we built a Bayesian classifier model and predicted a probabilistic functional relationship score for each isoform pair.

We first investigated the influence of the discriminativeness of the input data on the predictive performance of this algorithm at the isoform pair level. We simulated that, for both positive and negative isoform pairs, the input feature values follow a normal distribution, with standard deviation equal to 1. Then, the mean difference (MD) values between the positives and the negatives can vary with the discriminativeness of the feature. With MGR fixed at 0.3, the prediction accuracies at the isoform pair level in terms of AUC with MD = 0.1, 0.2 and 0.3 are shown in Fig. 2D–F. As expected, with increasing MD values the classification performance improved significantly. The median AUC on the 20 test sets for MD = 0.1, 0.2, 0.3 are 0.659, 0.838 and 0.929, respectively. In addition, we also calculated the AUPRC (area under precision recall curve) and found that it also improves with increasing MD values (Fig. 2G–I). This observation suggests that this algorithm works well with input genomic data of very weak (MD = 0.1 or 0.2) discriminative power.

**Figure 3:**
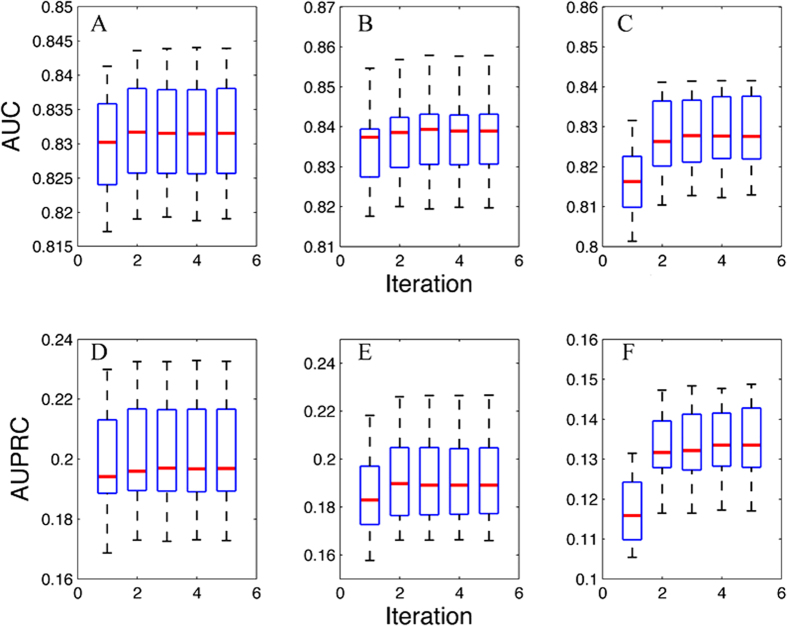
Isoform pair level predictive performance on the simulated data at different multi-isoform gene ratios (MGR). The mean difference of the input features between functional related and unrelated isoform pairs was fixed at 0.2 (see Fig. 2B). The MGR in A/D, B/E and C/F are 0.2, 0.3 and 0.5, respectively.

We further looked at how the predictive performance of this algorithm will change with the multi-isoform gene ratio. To this end, we fixed the value of the mean difference of input data to be 0.2. The prediction accuracy at the isoform level in terms of AUC at MGR = 0.2, 0.3 and 0.5 are 0.831, 0.838 and 0.827, respectively (Fig. 3A–C). This range of MGR is equivalent to the current MGR ratio in the RefSeq database. In addition, we also calculated the AUPRC (Fig. 3D–F), which is much higher than the baseline AUC (approximately 0.05) since the proportion of simulated functionally related gene pairs is 0.05. Interestingly, we found that the performance gain of the converged model over the model at the first iteration increases with the fraction of multi-isoform genes. These gains for MGR = 0.2, 0.3, 0.5 are 0.0019, 0.0030 and 0.0124 for AUC. Overall, this shows that this algorithm is robust against the percentage of multi-isoform genes among all genes, and will remain to be applicable when new alternatively spliced isoforms are identified and verified.

We also analyzed the effects of different combinations of MD and MGR values, and found that, for assigning isoform pair-level labels, this algorithm is robust to variations of the input data accuracy as well as the fraction of multi-isoform genes (Supplementary Figs S2–S4).

### Modeling and validating the functional relationship network using single-isoform gene pairs as true gold standard

The RefSeq gene build (version 37.2) was used in our study to build the isoform network for the mouse because it is validated and of high quality. First, we evaluated the performance of each type of feature data through 5-fold cross validation. The results in terms of AUC are presented in Table 1 (also Fig. S5). The AUC of all these features range from 0.521 to 0.612 with protein docking data ranking the highest. The reason why docking is most discriminating may be that it directly models protein binding, unlike expression features which captures only co-expression/co-regulation properties. The integrated network achieves the best performance, supporting expected power of integrated data analysis. The gold standard contains a large proportion of genes containing only a single isoform. Gene pairs formed by such single-isoform genes are therefore equal to isoform-level gold standard and therefore can be used for evaluating the performance of isoform networks.

**Figure 4:**
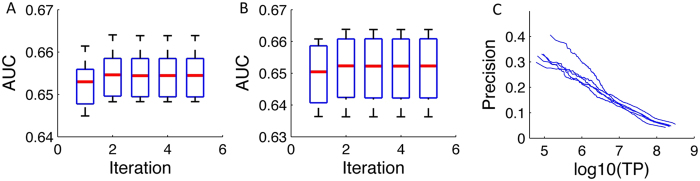
Predictive performance for the mouse functional relationship network based on 5-fold cross validation. Results are shown for single-isoform gene pairs (**A**) and multi-isoform gene pairs (**B**), separately. Precision-recall curves for single-isoform gene pairs are presented in (**C**).

To more reliably assess the performance on the real mouse data, only the experimentally validated (evidence codes: EXP, IMP, IPI, IGI, IEP and IDA) gene annotation in Gene Ontology (79,562 pairs) combined with those from KEGG and BioCyc pathways (106,276 positive pairs in total) were used for computational validation. We randomly generated 5 disjoint training and test sets for cross-validation, and showed the predictive performances of single-isoform gene pairs in Fig. 4A. The results from single-isoform gene pairs (true gold standard) are highly accurate with AUC being 0.656 ± 0.002, demonstrating that the proposed SIB-MIL method works very well also with experimental data. In addition, for multi-isoform gene pairs, we assigned each gene pair a score as the maximum probability of all its isoform pairs (since the isoform-level information is not available–unlike the simulation studies), under the assumption that co-functionality of a gene pair must be carried out by at least one of its isoform pairs. Gene-level prediction results are also provided in Fig. 4B, which are similar to that of single-isoform pairs. In addition, we also tested the performance of alternative validation methods using pre-filtered feature subsets (Fig. S6). These results are also accurate.

We further validated the predictions with a set of experimentally verified isoform-isoform interaction data provided in the Corominas data^23^. This dataset is recent, and it is the largest isoform-level, experimentally validated interaction dataset that has been generated. In this study, the authors tested a set of multi-isoform genes against a set of isoforms for interactions. As a result, for each interacting gene pair, one of the isoform pairs may be interacting, while others are not. The uniqueness of this dataset allows us to directly test whether we will be able to differentiate the isoform pairs that are truly interacting, against the isoform pairs that come from the same gene pair but are not interacting. Based on the supplementary materials of this paper, we obtained a total number of 629 isoform pairs that have interactions. Of the 629 pairs, 614 are between genes. These 614 pairs are used as positives. The remaining 15 pairs are interactions between the same isoforms and are excluded from this analysis. Next, we identified the gene pairs that contain these 614 isoform pairs. These gene pairs contained 1304 isoform pairs in total. This means that 690 isoform pairs are not identified as interacting in this experiment. These 690 isoform pairs are used as negatives. This set allows us to test whether we can differentiate which isoforms are actually interacting, against the isoform pairs coming from the same gene pair but not interacting. This is a desired and unique function of the algorithm described in this study.

**Figure 5:**
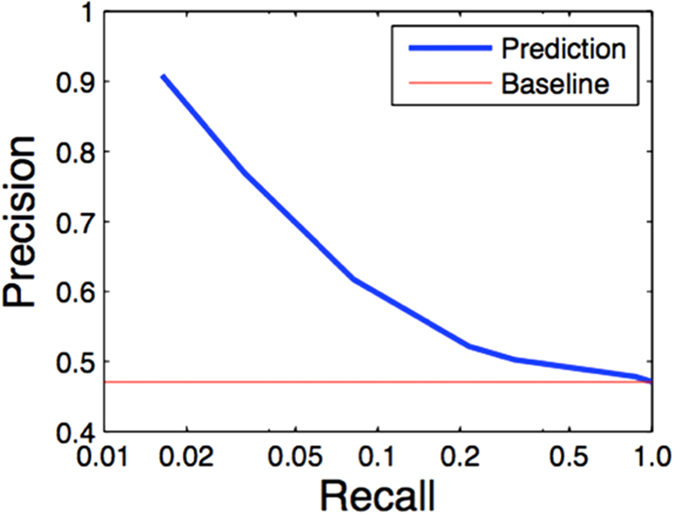
Experimental testing of the predictions using the validated isoform level protein-protein interaction data from Corominas, R. *et al. Nat Commun, 2014* (Ref. 23).

We found that our predictions are accurate with excellent precision for the top predictions (Fig. 5). The precision, obtained at the isoform level, is comparable to those of our previous studies obtained at the gene level^6^,^7^,^45^. This validates that even for the same gene pair, this method is now capable of differentiating the isoform pairs that are truly interacting, versus the ones that are not. This is a capability that is new to this method, and would be valuable to the mouse genetics community.

We have built a genome-wide isoform-level network for the mouse by integrating the isoform-level genomic features and the gene-level gold standard functionally related gene pairs. We also shuffled the gold standard and computed a random network whose isoform pair scores are closely normally distributed with mean 0.050 and standard deviation 0.023. Based on this randomization control, a score >0.119 (3 standard deviation away) in the network would be likely non-random.

We next evaluated the accuracy of the newly predicted highest scored isoform pairs (not recorded in GO, KEGG or BioCyc) against public databases, including protein-protein interactions^49^,^50^,^51^,^52^,^53^,^54^,^55^, MSigDB gene sets^56^ and Reactome pathways^29^ (Table 2). These databases provide a rich resource to test the performance of novel predictions. For multi-isoform gene pairs, we assigned the maximal probability of all its isoform pairs to each gene pair. We identified a list of 680, 624 gene pairs with probability >0.95, representing a set of functionally highly related gene pairs that were newly predicted. We found that 36.0% of top predicted gene pairs (244,957 gene pairs) were supported by co-annotation to the same biological process/pathway or having physical/genetic interactions, which is significant (*p* < 0.000001), compared to 7.0% of randomly generated gene pairs. This result further supports the high precision of the proposed network prediction model.

### The isoform-level network provides a high-resolution view of functional relationships

**Figure 6:**
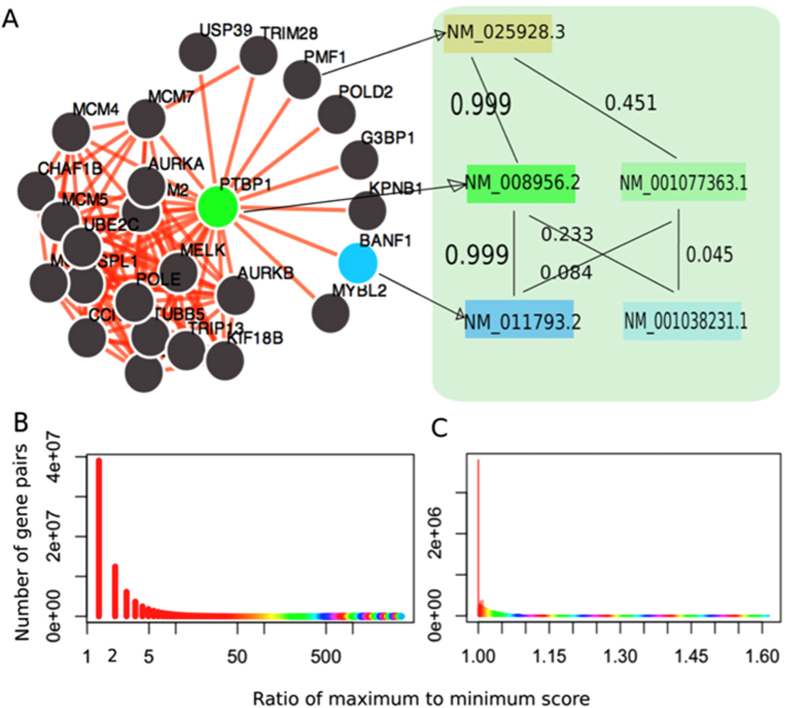
The isoform-level network reveals a high-resolution map of gene-gene co-functionality. (**A**) The left panel displays a traditional gene network of *Ptpb1*. The link between *Ptbp1* and *Banf1* can be dissected into 4 isoform-level linkages. (**B**) For each multi-isoform gene pair, we calculated the ratio of the maximum to minimum probability among all its isoform pairs as a measure of functional linkage diversity of different isoform pairs from the same gene pair. Shown here is the distribution of this ratio of 75, 512, 782 gene pairs. 25% of the gene pairs have a value larger than 3. (**C**) We also calculated the ratios of maximum to minimum score of multi-isoform gene pairs using a random network built with shuffled gold standard. The values fall into a short range from 0 to 1.60, suggesting the significance of functional diversity of different isoform pairs of a gene pair.

We found that this isoform-level functional relationship network for the mouse is capable of identifying differential functional relationships between isoform pairs belonging to the same gene pair. For example, the local gene-level network of *Ptbp1* (pyrimidine tract binding protein 1, an element of the spliceosome machinery) describes the functional relationship between any two genes with a single probability value (Fig. 6A, left panel). In fact, many genes in this network have multiple isoforms. For example, both *Ptbp1* and *Banf1* have two alternatively spliced isoforms. The functional relationship between *Ptbp1* and *Banf1* in this isoform-level network can therefore be dissected into four functional linkages corresponding to four isoform pairs (Fig. 6A, right panel). Among the four isoform pairs, the functional relationship of the isoform pair [*NM_008956.2*, *NM_011793.2*] is predicted to be 0.999, whereas the probabilities of the other three isoform pairs are much lower −0.233, 0.084, and 0.045, respectively.

Such disparities of connections between isoform pairs of the same gene pair are prevalent. To quantify such differences, we calculated the ratio of the maximum to the minimum of the predicted probability among all isoform pairs of a given gene pair, respectively. For example, this ratio for the *Ptbp1* and *Banf1* gene pair is calculated as 0.999/0.045 = 22.2, where 0.999 and 0.045 are the maximum and minimum score between this gene pair, respectively (Fig. 6A). From the whole isoform-level network of the mouse, we found that this ratio spans a wide range from 1.0 (no difference) to more than 3500 (3500 fold difference) (Fig. 6B). Notably, 25% of these gene pairs have a fold change value larger than 3.0, implying that a high proportion of the gene pairs are functionally highly differentiated at the isoform level. The significance of the wide ratio span was indicated using a random network built with shuffled gold standard (Fig. 6C). These results suggest that difference is prevalent across isoform pairs coming from the same gene pair and that this isoform network can reveal such variations.

### The isoform-level network reveals functional diversity of different isoforms of the same gene

It is known that proteins encoded by isoforms of the same gene can carry out different and even opposite biological functions, such as pro-apoptotic versus anti-apoptotic actions of bclx-L vs bclx-S and of caspase 3 (L vs S) and transcriptional activation versus transcriptional repression for odd-skipped 2^34^. Investigating and revealing the functional diversity of the same gene achieved by alternative splicing is pivotal to biology. Because of its high resolution, this isoform network has the ability to reveal such functional diversity.

**Figure 7:**
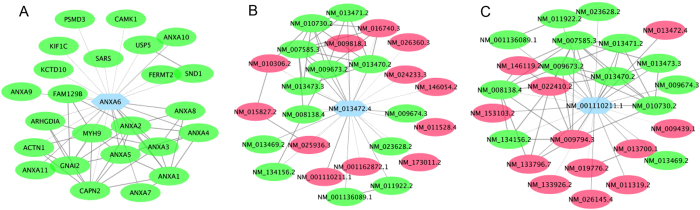
(**A**) The gene-level functional relationship network of the *Anxa6* gene. (**B**,**C)** show the isoform-level networks of its two isoforms: NM_013472.4 and NM_001110211.1, respectively. Red nodes in (**B**,**C)** denote the neighboring isoforms not shared by the two isoforms. The networks of the two isoforms reveal different connections reflecting their respective functional roles.

To systematically examine the functional diversity represented at the network level, for each of the 3427 validated multi-isoform genes in the RefSeq database, we compared the networks (with the top 25 neighbors) of its isoforms and counted the number of shared functionally related neighbors (Supplementary Fig. S7). We found that the minimum, mean and maximum numbers of shared neighbors are 0, 4 and 24, respectively. These statistics indicate that many isoforms of the same gene have different functional connections and may participate in different biological processes. Based on literature^15^,^30^,^34^,^47^, we collected a list of genes whose isoforms were shown to have different functions, and calculated for each gene the number of shared connections between isoform networks (Table 3). For example, *Anxa6* has two alternatively spliced isoforms: NM_001110211.1 and NM_013472.4. Both isoforms have the same N- and C-termini, but the former encodes a shorter protein by six amino acids (525–530) due to the lack of an alternate in-frame exon compared to the latter. As an illustration, we identified the local networks of these two isoforms (Fig. 7). Their local networks share only 13 out of 25 neighboring isoforms, indicating a diverse functional relationship map of these two isoforms despite similar structures.

To investigate the functional differences of the genes in the two local networks, we performed Gene Ontology (biological process terms) enrichment analysis using the top connected genes in each network (Supplementary Table S2). We found that, while sharing the same GO terms (such as GO:0006944 cellular membrane fusion and GO:0061025 membrane fusion), the two isoform networks are also enriched for genes annotated to different GO biological processes. The isoform network of NM_001110211.1 is enriched for genes associated to vesicle fusion (GO:0006906, *p* = 0.0096), organelle fusion (GO:0048284, *p* = 0.044), and amino acid activation (GO:0043038, *p* = 0.0262), whereas the isoform network of NM_013472.4 is enriched for genes related to regulation of cell shape (GO:0008360, *p* = 0.0135). These disparate enriched functions strongly support the functional diversity of the two isoforms of the *Anxa6* gene. This computational modeling of the folding and conformation of the two isoforms shows a striking difference in likelihood of phosphorylation in the Thr-Pro-Ser (535–537 vs 529–531) sequence^34^. In addition, the alternative splice isoforms of the *Anxa6* gene have been reported to have functional differences on catecholamine secretion^57^, which is consistent with this functional enrichment analysis related to the vesicle fusion and organelle fusion (Supplementary Table S2). These results suggest that this isoform-level network is able to reveal functional diversity of different isoforms of the same gene and could therefore become a promising tool for investigating gene functions at the isoform level. To facilitate new isoform function annotation based on this network, we included this enrichment analysis for all local networks of individual isoforms in our website.

## Discussion

We have developed a novel Bayesian network-based multiple instance learning approach to probe functional relationships at the isoform level, thus being able to provide a higher resolution view compared to traditional gene-level networks. Determining functional connections between splice isoforms from the same gene is essential to functional genomics, which would help deepen our understanding of gene functions and functional relationships and may provide useful information on diseases caused by alternative splicing. It is widely understood that the topology of molecular pathways varies between transcripts or protein isoforms. The current work also has limitations since it represents a generic network without considering tissue-specific expression of isoforms across tissues or cell types. A major next step is to build tissue, cell-type, and phenotype-specific networks for more refined understanding of functional relationships in a given context both for the mouse and for the extension of this strategy into human studies. As an example, isoform networks for normal brain and brain with Alzheimer’s disease (AD) would be of interest for understanding perturbed pathways or networks in AD and probably heterogeneous causes of AD. The established isoform network provides a first systematic attempt to convey isoform-level connection and functional relationships. We expect that isoform-level networks will find wide applications in genomic and biomedical applications, and that the current gene-centered network modeling approach will be expanded to a more refined isoform level.

## Additional Information

**How to cite this article**: Li, H.-D. *et al.* A Network of Splice Isoforms for the Mouse. *Sci. Rep.* **6**, 24507; doi: 10.1038/srep24507 (2016).

## Supplementary Material

Supplementary Information

## Figures and Tables

**Table 1:** Comparison of 5-fold cross-validated prediction for each type of feature data and the integrated network.

Data	RNA-seq	Exon array	Pseudo-AAC	Protein-docking	Integrated
AUC	0.535 ± 0.0137	0.521 ± 0.0083	0.575 ± 0.0004	0.612 ± 0.0013	0.624 ± 0.0001

**Table 2:** Validation results of the novel predictions of highly related gene pairs against pathway and interaction databases.

Database	Validated	Random	Fold change
Protein-Protein Interactions	350	26 ± 5	13.5
MSigDB gene sets	243447	4308 ± 199	5.7
Reactome pathways	24508	1430 ± 38	17
Total	244957	43246 ± 198	5.6

The novel predictions (680,624 gene pairs with a predicted probability >0.95) excluded those that were initially used in gold standard. The number of validated pairs is significant (p < 0.000001) based on 1000 times of randomly generated gene pairs. The protein-protein interaction data were collected from the MINT^52^, MIPS^49^, DIP^58^, IntAct^55^ and BioGRID^51^ database.

**Table 3:** Example genes whose isoforms share no or few connections in their networks based on the predicted mouse isoform networks.

Gene	Isoforms A	Isoform B	N_share_
*Anxa6*	NM_001110211.1	NM_013472.4	13
*Cdkn2a*	NM_009877.2	NM_001040654.1	4
*Calu*	NM_184053.2	NM_007594.3	11
*Cflar*	NM_207653.3	NM_009805.4	10
*Lmna*	NM_001111102.1	NM_019390.2	9
	NM_001111102.1	NM_001002011.2	15
	NM_019390.2	NM_001002011.2	9
*Egfr*	NM_007912.4	NM_207655.2	7
*Ptbp1*	NM_008956.2	NM_001077363.1	1
*Ola1*	NM_025942.2	NM_030091.1	6
*Mkl1*	NM_153049.2	NM_001082536.1	3
*Tufm*	NM_172745.3	NM_001163713.1	3

N_share_ is the number of shared connections out of the top 25 connections.

## References

[b1] Lee I., Date S. V., Adai A. T. & Marcotte E. M. A probabilistic functional network of yeast genes. Science 306, 1555–1558 (2004). 15567862 10.1126/science.1099511

[b2] Franke L. *et al.* Reconstruction of a functional human gene network, with an application for prioritizing positional candidate genes. Am. J. Hum. Genet. 78, 1011–1025 (2006). 16685651 10.1086/504300 PMC1474084

[b3] Guan Y. *et al.* A genomewide functional network for the laboratory mouse. Plos Comput. Biol. 4, e1000165 (2008). 18818725 10.1371/journal.pcbi.1000165 PMC2527685

[b4] Hwang S., Rhee S. Y., Marcotte E. M. & Lee I. Systematic prediction of gene function in Arabidopsis thaliana using a probabilistic functional gene network. Nat. Protoc. 6, 1429–1442 (2011). 21886106 10.1038/nprot.2011.372 PMC3654671

[b5] Karaoz U. *et al.* Whole-genome annotation by using evidence integration in functional-linkage networks. Proc. Natl. Acad. Sci. USA 101, 2888–2893 (2004). 14981259 10.1073/pnas.0307326101 PMC365715

[b6] Guan Y., Ackert-Bicknell C. L., Kell B., Troyanskaya O. G. & Hibbs M. A. Functional genomics complements quantitative genetics in identifying disease-gene associations. Plos Comput. Biol. 6, e1000991 (2010). 21085640 10.1371/journal.pcbi.1000991 PMC2978695

[b7] Guan Y. *et al.* Tissue-specific functional networks for prioritizing phenotypes and disease genes. Plos Comput. Biol. 8, e1002694 (2012). 23028291 10.1371/journal.pcbi.1002694 PMC3459891

[b8] Recla J. M. *et al.* Precise genetic mapping and integrative bioinformatics in diversity outbred mice reveals Hydin as a novel pain gene. Mamm. Genome 25, 211–222 (2014). 24700285 10.1007/s00335-014-9508-0 PMC4032469

[b9] Zhao X.-M., Wang Y., Chen L. & Aihara K. Protein domain annotation with integration of heterogeneous information sources. Proteins: Struct. Funct. Bioinf. 72, 461–473 (2008). 10.1002/prot.21943 18214951

[b10] Liu Z.-P., Wu L.-Y., Wang Y., Chen L. & Zhang X.-S. Predicting gene ontology functions from protein’s regional surface structures. BMC Bioinformatics 8, 1–13 (2007). 18070366 10.1186/1471-2105-8-475 PMC2233648

[b11] Yalamanchili H. K. *et al.* SpliceNet: recovering splicing isoform-specific differential gene networks from RNA-Seq data of normal and diseased samples. Nucleic Acids Res. 42, e121 (2014). 25034693 10.1093/nar/gku577 PMC4150760

[b12] Tseng Y.-T. *et al.* IIIDB: a database for isoform-isoform interactions and isoform network modules. BMC Genomics 16, S10 (2015). 25707505 10.1186/1471-2164-16-S2-S10 PMC4331710

[b13] Li W., Dai C., Kang S. & Zhou X. J. Integrative analysis of many RNA-seq datasets to study alternative splicing. Methods 67, 313–324 (2014). 24583115 10.1016/j.ymeth.2014.02.024 PMC4120771

[b14] Li W. *et al.* High-resolution functional annotation of human transcriptome: predicting isoform functions by a novel multiple instance-based label propagation method. Nucleic Acids Res. 1–15 (2013). 24369432 10.1093/nar/gkt1362 PMC3973446

[b15] Li H.-D., Menon R., Omenn G. & Guan Y. The emerging era of genomic data integration for analyzing splice isoform functions. Trends Genet. 30, 340–347 (2014). 24951248 10.1016/j.tig.2014.05.005 PMC4112133

[b16] Omenn G. S., Menon R. & Zhang Y. Innovations in proteomic profiling of cancers: Alternative splice variants as a new class of cancer biomarker candidates and bridging of proteomics with structural biology. J. Proteomics 90, 28–37 (2013). 23603631 10.1016/j.jprot.2013.04.007 PMC3841011

[b17] Trapnell C. *et al.* Differential gene and transcript expression analysis of RNA-seq experiments with TopHat and Cufflinks. Nat. Protoc. 7, 562–578 (2012). 22383036 10.1038/nprot.2012.016 PMC3334321

[b18] Feng J., Li W. & Jiang T. Inference of isoforms from short sequence reads. J. Comput. Biol. 18, 305–321 (2011). 21385036 10.1089/cmb.2010.0243 PMC3123862

[b19] Mortazavi A., Williams B. A., McCue K., Schaeffer L. & Wold B. Mapping and quantifying mammalian transcriptomes by RNA-Seq. Nat. Methods 5, 621–628 (2008). 18516045 10.1038/nmeth.1226 PMC13303166

[b20] Wang Z., Gerstein M. & Snyder M. RNA-Seq: a revolutionary tool for transcriptomics. Nat. Rev. Genet. 10, 57–63 (2009). 19015660 10.1038/nrg2484 PMC2949280

[b21] Guerler A., Govindarajoo B. & Zhang Y. Mapping monomeric threading to protein-protein structure prediction. J. Chem. Inf. Model. 53, 717–725 (2013). 23413988 10.1021/ci300579r PMC4076494

[b22] Ellis Jonathan D. *et al.* Tissue-specific alternative splicing remodels protein-protein interaction networks. Mol. cell 46, 884–892 (2012). 22749401 10.1016/j.molcel.2012.05.037

[b23] Corominas R. *et al.* Protein interaction network of alternatively spliced isoforms from brain links genetic risk factors for autism. Nat. Commun. 5, 3650 (2014). 24722188 10.1038/ncomms4650 PMC3996537

[b24] Ashburner M. *et al.* Gene Ontology: tool for the unification of biology. Nat. Genet. 25, 25–29 (2000). 10802651 10.1038/75556 PMC3037419

[b25] Kanehisa M., Goto S., Kawashima S., Okuno Y. & Hattori M. The KEGG resource for deciphering the genome. Nucleic Acids Res. 32, D277–D280 (2004). 14681412 10.1093/nar/gkh063 PMC308797

[b26] Kanehisa M., Goto S., Furumichi M., Tanabe M. & Hirakawa M. KEGG for representation and analysis of molecular networks involving diseases and drugs. Nucleic Acids Res. 38, D355–360 (2010). 19880382 10.1093/nar/gkp896 PMC2808910

[b27] Karp P. D. *et al.* Expansion of the BioCyc collection of pathway/genome databases to 160 genomes. Nucleic Acids Res. 33, 6083–6089 (2005). 16246909 10.1093/nar/gki892 PMC1266070

[b28] Joshi-Tope G. *et al.* Reactome: a knowledgebase of biological pathways. Nucleic Acids Res. 33, D428- 432 (2005). 15608231 10.1093/nar/gki072 PMC540026

[b29] Jupe S., Akkerman J. W., Soranzo N. & Ouwehand W. H. Reactome - a curated knowledgebase of biological pathways: megakaryocytes and platelets. J. Thromb. Haemost. 10, 2399–2402 (2012). 22985186 10.1111/j.1538-7836.2012.04930.x PMC3578965

[b30] Eksi R. *et al.* Systematically differentiating functions for alternatively spliced isoforms through integrating RNA-seq data. Plos Comput. Biol. 9, e1003314 (2013). 24244129 10.1371/journal.pcbi.1003314 PMC3820534

[b31] Omenn G. S., Guan Y. & Menon R. A new class of protein cancer biomarker candidates: Differentially expressed splice variants of ERBB2 (HER2/neu) and ERBB1 (EGFR) in breast cancer cell lines. J. Proteomics 107, 103–112 (2014). 24802673 10.1016/j.jprot.2014.04.012 PMC4123867

[b32] Li H.-D. *et al.* Modeling the functional relationship network at the splice isoform level through heterogeneous data integration. *bioRxiv*, doi: 10.1101/001719 (2013).

[b33] Aranda B. *et al.* The IntAct molecular interaction database in 2010. Nucleic Acids Res 38, D525–531 (2010). 19850723 10.1093/nar/gkp878 PMC2808934

[b34] Menon R. *et al.* Functional implications of structural predictions for alternative splice proteins expressed in Her2/neu-induced breast cancers. J. Proteome Res. 10, 5503–5511 (2011). 22003824 10.1021/pr200772w PMC3230717

[b35] Andrews S., Tsochantaridis I. & Hofmann T. Support vector machines for multiple-instance learning. In *Neural Inf. Process Syst.* (2003).

[b36] Li H.-D., Liang Y.-Z. & Xu Q.-S. Support vector machines and its applications in chemistry. Chemometr. Intell. Lab. Syst. 95, 188–198 (2009).

[b37] Li H.-D. *et al.* Recipe for uncovering predictive genes using support vector machines based on model population analysis. IEEE/ACM T Comput Bi 8, 1633–1641 (2011). 10.1109/TCBB.2011.36 21339535

[b38] Troyanskaya O. G., Dolinski K., Owen A. B., Altman R. B. & Botstein D. A Bayesian framework for combining heterogeneous data source for gene function prediction (in Saccharomyces cerevisiae). Proc. Natl Acad. Sci. USA 100, 8348–8353 (2003). 12826619 10.1073/pnas.0832373100 PMC166232

[b39] Huttenhower C., Hibbs M., Myers C. & Troyanskaya O. G. A scalable method for integration and functional analysis of multiple microarray datasets. Bioinformatics 22, 2890–2897 (2006). 17005538 10.1093/bioinformatics/btl492

[b40] Pop A., Huttenhower C., Iyer-Pascuzzi A., Benfey P. & Troyanskaya O. Integrated functional networks of process, tissue, and developmental stage specific interactions in Arabidopsis thaliana. BMC Syst. Biol. 4, 180 (2010). 21194434 10.1186/1752-0509-4-180 PMC3023688

[b41] Huttenhower C. *et al.* Exploring the human genome with functional maps. Genome Res. 19, 1093–1106 (2009). 19246570 10.1101/gr.082214.108 PMC2694471

[b42] Wong A. K. *et al.* IMP: a multi-species functional genomics portal for integration, visualization and prediction of protein functions and networks. Nucleic Acids Res. 40, W484–W490 (2012). 22684505 10.1093/nar/gks458 PMC3394282

[b43] Park C. Y. *et al.* Functional knowledge transfer for high-accuracy prediction of under-studied biological processes. Plos Comput. Biol. 9, e1002957 (2013). 23516347 10.1371/journal.pcbi.1002957 PMC3597527

[b44] Wang Y., Zhang X.-S. & Xia Y. Predicting eukaryotic transcriptional cooperativity by Bayesian network integration of genome-wide data. Nucleic Acids Res 37, 5943–5958 (2009). 19661283 10.1093/nar/gkp625 PMC2764433

[b45] Guan Y. *et al.* Predicting gene function in a hierarchical context with an ensemble of classifiers. Genome Biol. 9 Suppl 1, S3 (2008). 10.1186/gb-2008-9-s1-s3 PMC2447537 18613947

[b46] Yanai I. & DeLisi C. The society of genes: networks of functional links between genes from comparative genomics. Genome Biol. 3, research0064.0061-research0064.0012 (2002). 10.1186/gb-2002-3-11-research0064 PMC133448 12429063

[b47] Li H.-D. *et al.* Functional networks of highest-connected splice isoforms: from the Chromosome 17 Human Proteome Project. J. Proteome Res. 14, 3484–3491 (2015). 26216192 10.1021/acs.jproteome.5b00494 PMC4993635

[b48] Pan Q., Shai O., Lee L. J., Frey B. J. & Blencowe B. J. Deep surveying of alternative splicing complexity in the human transcriptome by high-throughput sequencing. Nat Genet 40, 1413–1415 (2008). 18978789 10.1038/ng.259

[b49] Mewes H. W. *et al.* MIPS: a database for genomes and protein sequences. Nucleic Acids Res. 30, 31–34 (2002). 11752246 10.1093/nar/30.1.31 PMC99165

[b50] Mewes H. W. *et al.* MIPS: analysis and annotation of proteins from whole genomes in 2005. Nucleic Acids Res. 34, D169–D172 (2006). 16381839 10.1093/nar/gkj148 PMC1347510

[b51] Stark C. *et al.* The BioGRID Interaction Database: 2011 update. Nucleic Acids Res. 39, D698–704 (2011). 21071413 10.1093/nar/gkq1116 PMC3013707

[b52] Licata L. *et al.* MINT, the molecular interaction database: 2012 update. Nucleic Acids Res. 40, D857–D861 (2012). 22096227 10.1093/nar/gkr930 PMC3244991

[b53] Zanzoni A. MINT: a Molecular INTeraction database. FEBS Lett. 513, 135–140 (2002). 11911893 10.1016/s0014-5793(01)03293-8

[b54] Xenarios I. *et al.* DIP, the Database of Interacting Proteins: a research tool for studying cellular networks of protein interactions. Nucleic Acids Res. 30, 303–305 (2002). 11752321 10.1093/nar/30.1.303 PMC99070

[b55] Hermjakob H. IntAct: an open source molecular interaction database. Nucleic Acids Res. 32, D452–D455 (2004). 14681455 10.1093/nar/gkh052 PMC308786

[b56] Subramanian A. *et al.* Gene set enrichment analysis: A knowledge-based approach for interpreting genome-wide expression profiles. Proc. Natl. Acad. Sci. USA 102, 15545–15550 (2005). 16199517 10.1073/pnas.0506580102 PMC1239896

[b57] Podszywalow-Bartnicka P. *et al.* Role of annexin A6 Isoforms in catecholamine secretion by PC12 cells: distinct influence on calcium response. J. Cell Biochem. 111, 168–178 (2010). 20506562 10.1002/jcb.22685

[b58] Deane C. M., Salwinski L., Xenarios I. & Eisenberg D. Protein interactions: two methods for assessment of the reliability of high-throughput observations. Mol. Cell Proteomics 1, 349– 356 (2002). 12118076 10.1074/mcp.m100037-mcp200

