# Supplementary material for: A Network of Splice Isoforms for the Mouse
Source: Sci Rep. 2016 Apr 15;6:24507. doi: 10.1038/srep24507 (PMC4832266; doi:10.1038/srep24507)
Supplement: Supplementary Information [file srep24507-s1.doc]

A Network of Splice Isoforms for the Mouse

Hong-Dong Li^1,2^, Rajasree Menon^1^, Ridvan Eksi^1^, Aysam Guerler^1^, Yang Zhang^1^, Gilbert S. Omenn^1,3,*^, Yuanfang Guan ^1,3,4*^

1. Department of Computational Medicine and Bioinformatics, University of Michigan, Ann Arbor, Michigan, United States
2. Institute for Systems Biology, Seattle, Washington, United States
3. Department of Internal Medicine, University of Michigan, Ann Arbor, Michigan, United States
4. Department of Electrical Engineering and Computer Science, Ann Arbor, Michigan, United States

*. Correspondence and requests for materials should be addressed to GSO (email: [gomenn@med.umich.edu](mailto:gomenn@med.umich.edu)) or YG (email: [gyuanfan@umich.edu](mailto:gyuanfan@umich.edu))

**Content:**

| **ID** | **Data** | **Description** |
| --- | --- | --- |
| 1 | **Figure S1** | Comparison of different MIL algorithms. |
| 2 | **Figure S2** | AUC of simulated data |
| 3 | **Figure S3** | AUPRC of simulated data |
| 4 | **Figure S4** | Precision-recall curves of the simulated data. |
| 5 | **Figure S5** | The prediction accuracy of each type of feature data. |
| 6 | **Figure S6** | Prediction performance of SIB-MIL with selected feature data using 20% randomly selected gold standard. |
| 7 | **Figure S7** | Distribution of the number of shared neighbors between any two isoforms of multi-isoform genes. |
| 8 | **Text S1** | Methods for processing isoform level genomic data and constructing gold standard gene pairs. |
| 9 | **Text S2** | Methods for data simulation. |
| 10 | **Text S3** | Significance test on experimentally validated data. |
| 11 | **Table S1** | Integrated genomic data for mouse isoform network. |
| 12 | **Table S2** | Gene Ontology enrichment of the isoforms of *Anxa6* gene. |


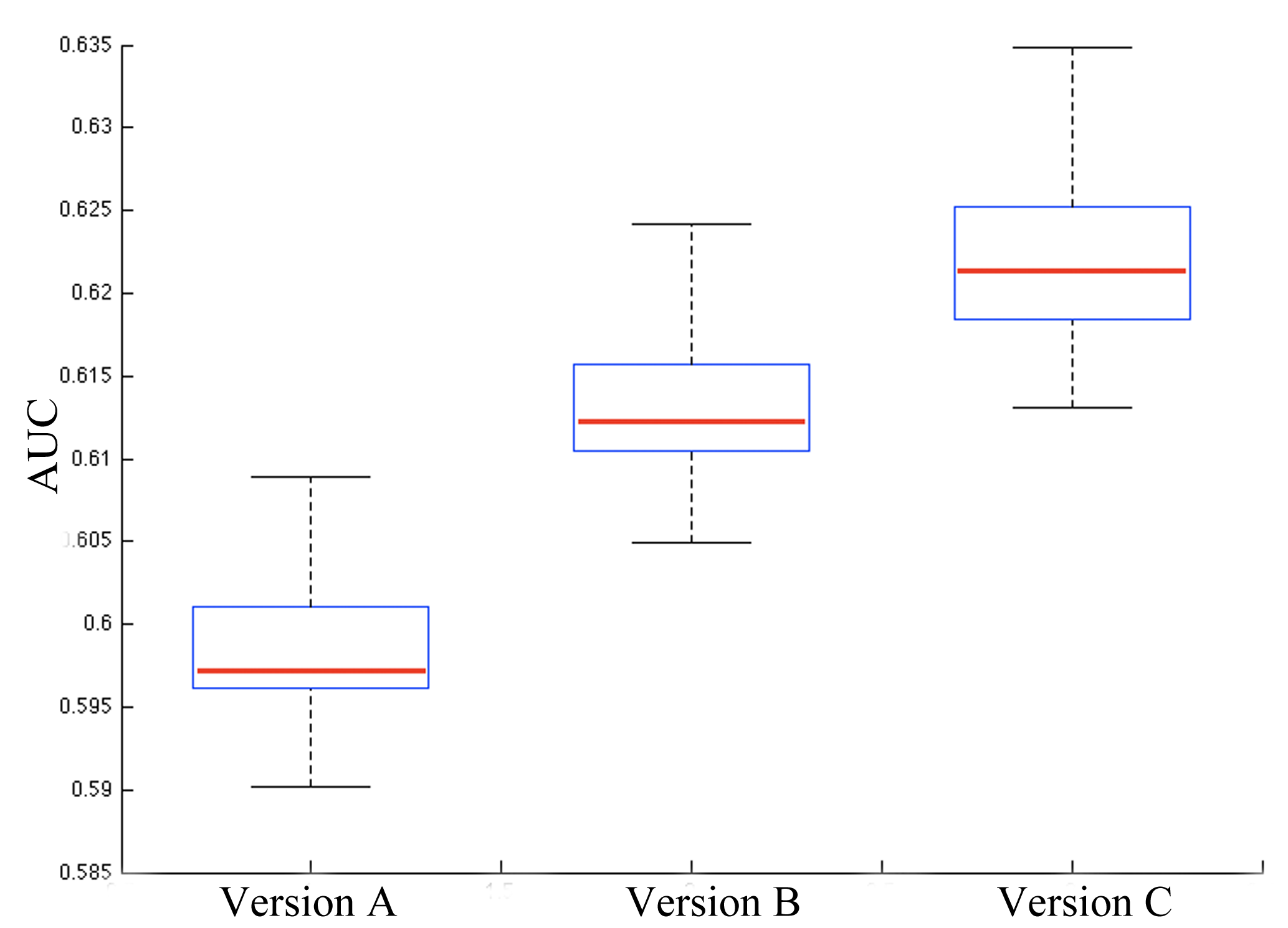


**Figure S1.** **Performance comparison of different MIL algorithms in terms of ROC curves computed on 20 randomly generated test sets**. Version A: the MI-SVM algorithm proposed in the work [^1^](#_ENREF_1) where a randomly selected isoform pair from gene pair bag is used as “witness” in its first iteration. Version B: a test version of MIL developed in our study whose initialization step is the same as that in Version A. From the second iteration, a subset of isoform pairs from negative gene pair bags were selected so as to keep the ratio of negative to positive isoform pairs the same as that in the first iteration. Version C: This method.


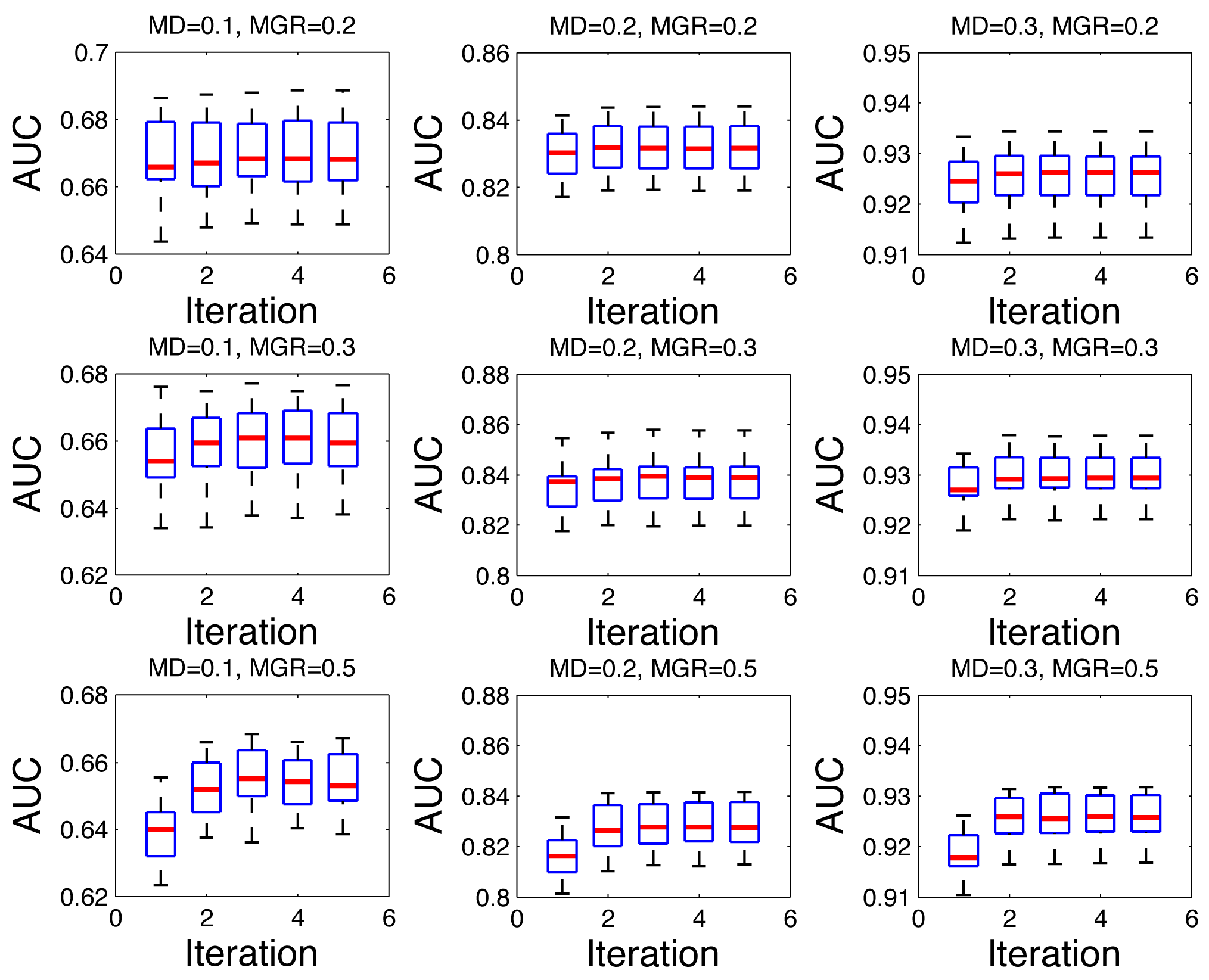


**Figure S2**. Performance (in AUCs) on the simulated data, with 9 settings of MD and MGR values.


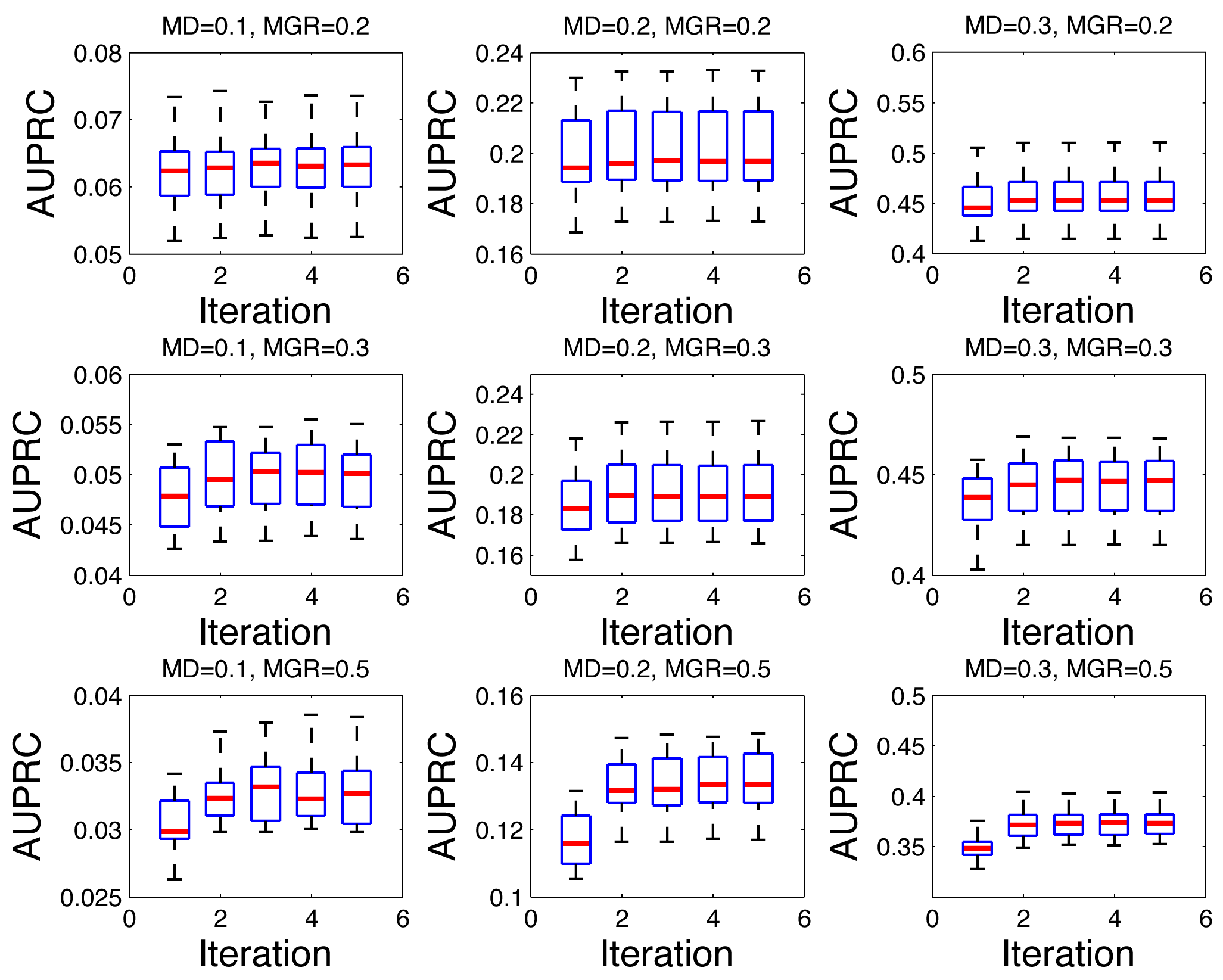


**Figure S3**. Performance (in AUPRCs) on the simulated data, with 9 settings of MD and MGR values.


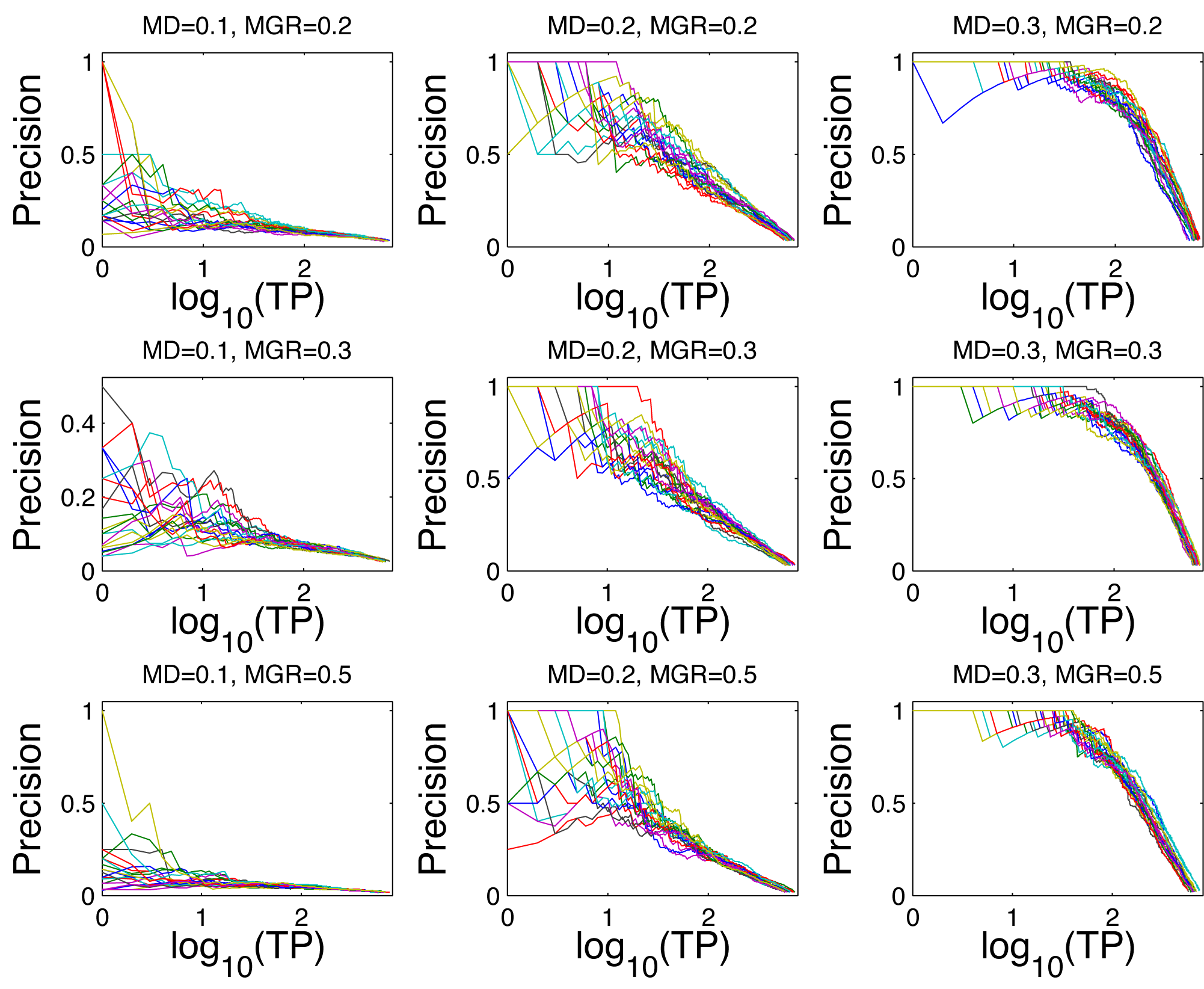


**Figure S4**. Performance (in precision-recall curves) on the simulated data, with 9 settings of MD and MGR values.


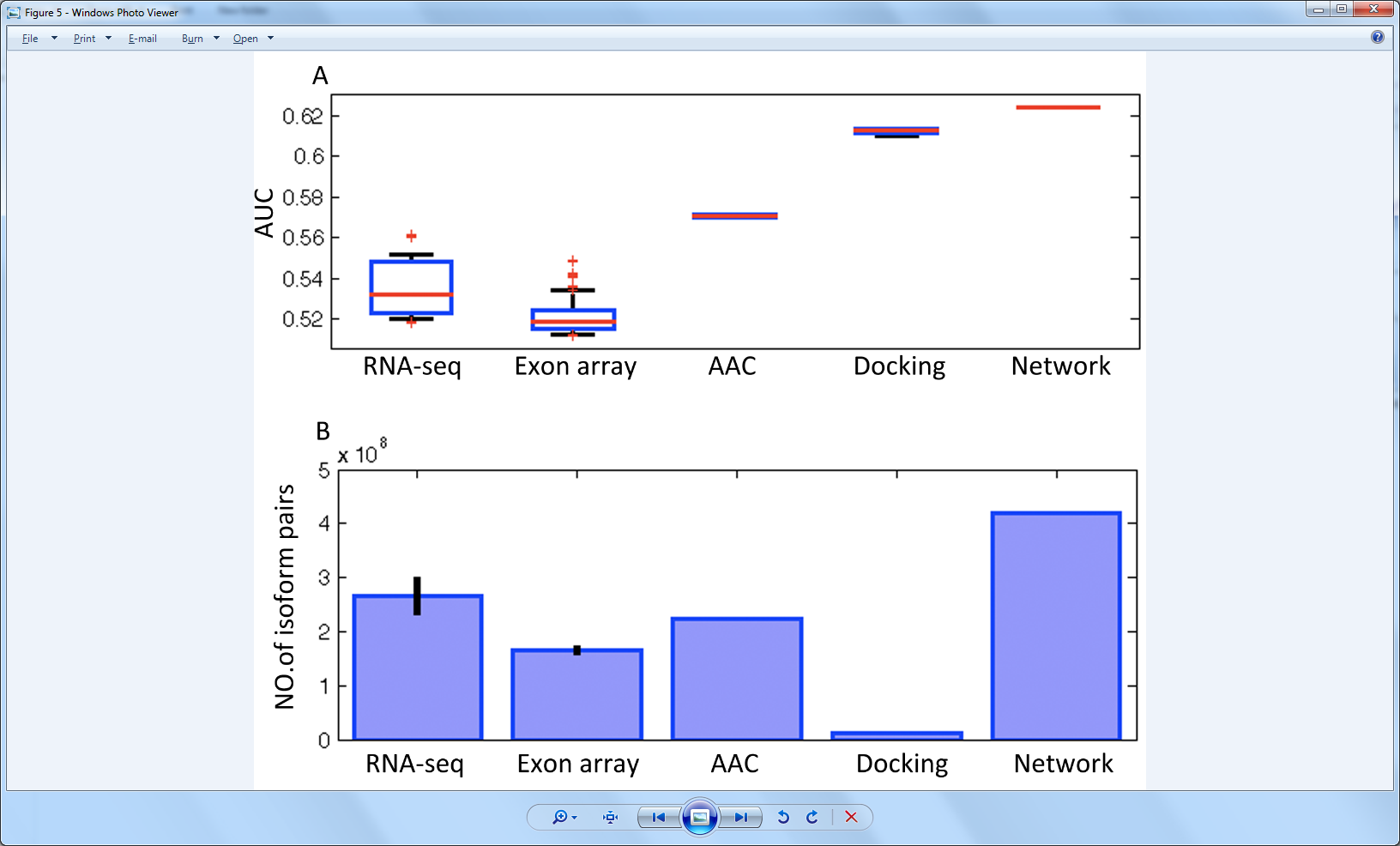


**Figure S5**. The prediction accuracy of each type of based on single-isoform gene pairs.


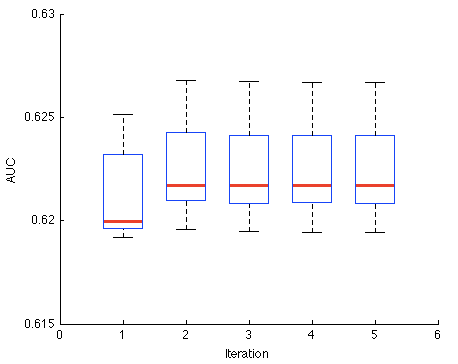


**Figure S6**. 20% of the gold standard was randomly chosen to select a subset of feature datasets. The remaining 80% were hold out and used to evaluate the predictive performance of the SIB-MIL algorithm on the mouse data.


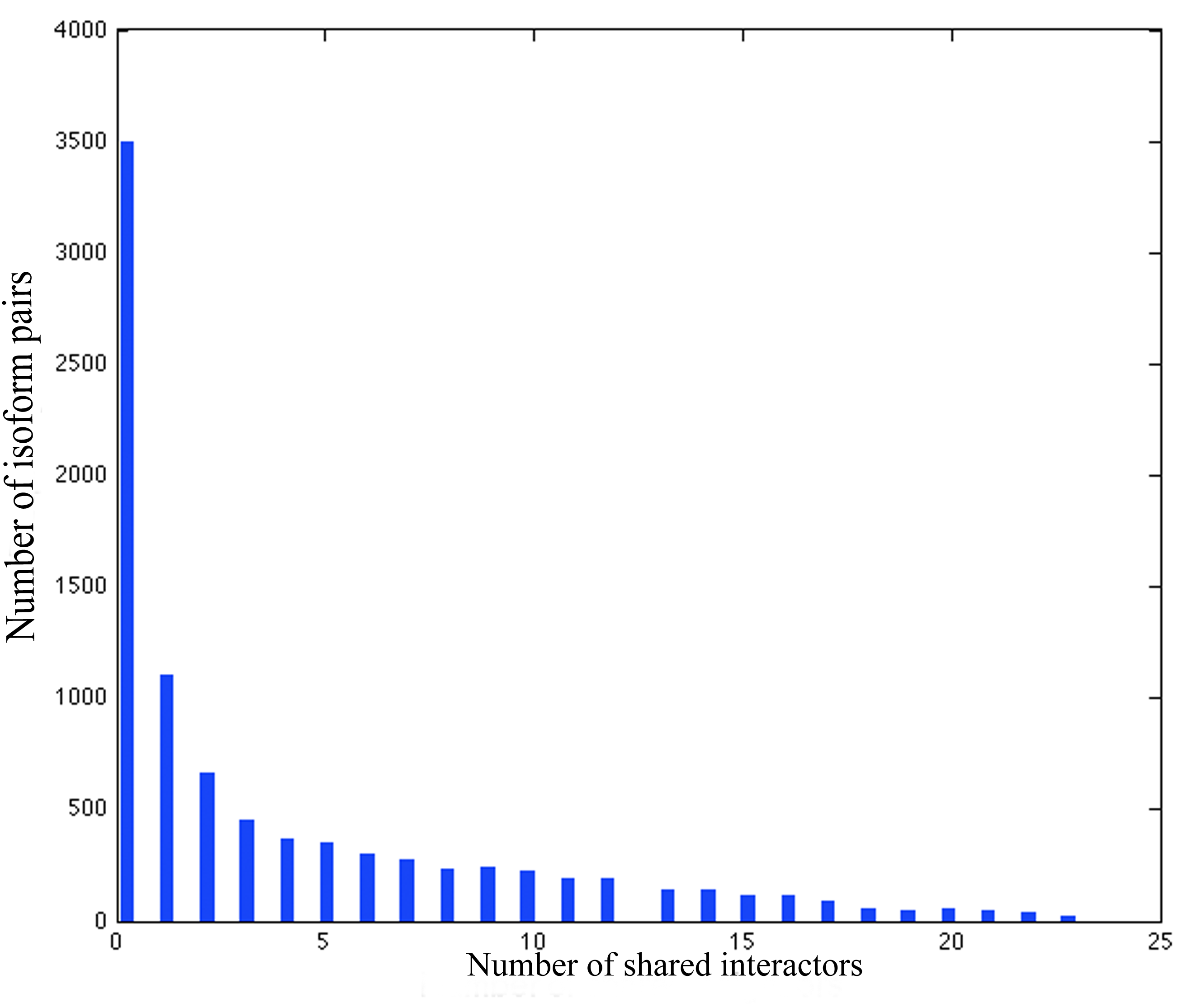


**Figure S7.** The distribution of the number of shared interactors (out of the top 25 interactors) between any two isoforms of the multi-isoform genes.

**Text S1. Isoform-level genomic data processing and gold standard construction of the mouse**

Initially, we had in total 164 isoform-level features: 41 from RNA-seq data, 121 from exon array, 1 from pseudo-amino acid composition and 1 from protein-docking score data. Details for processing these four types of data are described below. Protein domain data was excluded due to direct Gene Ontology annotation transfer from domain information in mouse.

***RNA-seq***. We downloaded 117 mouse RNA-seq datasets (corresponding to 811 experiments) from the NCBI sequence read archive (SRA) [^2^](#_ENREF_2) on May 1, 2012, which cover a wide range of experimental conditions and different tissues. For each RNA-seq experiment, we used the TopHat (v2.0.051) to align the reads against the *Mus Musculus* reference genome from the NCBI gene build (version 37.2). Then, the resulting mapped read files together with the corresponding transcript annotation files were processed by Cufflinks (v2.0.0) [^4^](#_ENREF_4) to calculate the relative abundance of the transcripts in terms of FPKM (Fragments Per Kilobase of exon per Million fragments). We removed those experiments with less than 10 million reads or covering less than 50% of the genes. In addition, to calculate correlations, those datasets with fewer than 4 experiments were also removed. In doing so, we finally obtained 41 datasets including 386 experiments. Within each dataset, we further removed those transcripts with more than 50% missing values to ensure the accuracy of expression correlation estimation. FPKM values were log-2 transformed as they were treated in Cuffdiff 2. We calculated Pearson correlation coefficients, denoted by *ρ*, between all possible transcript pairs for each dataset, followed by normalization using Fisher’s z-transformation [^5^](#_ENREF_5) to allow comparison between different datasets:

$z=\log(\frac{1-\rho}{1+\rho})$ (1)

This *z*-transformed correlation will be used as isoform-level features when building the Bayesian classifiers through the multiple instance learning approach.

***Exon array data***. 121 mouse exon array (Affymetrix Exon 1.0 ST array) datasets of the mouse were downloaded from the NCBI GEO (Gene Expression Omnibus) database. Each dataset includes at least 4 experiments. We calculated the expression of transcripts by utilizing the R package MEAP (version 2.0.1) [^6^](#_ENREF_6). Then, The Pearson correlation coefficient between each pair of transcripts was computed and normalized using the Formula (3). These correlations will be used as the feature inputs.

***Pseudo-amino acid composition***. Pseudo-amino acid composition (pseAAC) is a descriptor that characterizes the standard amino acid composition (AAC) as well as the pseudo-AAC by taking the sequence information of a protein into account [^7^](#_ENREF_7). Here, we calculated pseudo-amino acid composition (pseAAC) data for the protein-coding isoforms. The number of pseudo components was set to be 20. So each protein sequence was characterized by a 40 dimensional vector (composition of 20 natural amino acids plus 20 pseudo AACs). Then we calculated Fisher’s *z*-transformed Pearson correlation between isoform pairs as the feature data.

***Protein docking score***. We computed and derived a quantitative physical interaction score for each isoform pair using the SPRING algorithm [^8^](#_ENREF_8). Briefly, SPRING is a template-based algorithm for protein-protein structure prediction. It first threads one chain of the protein complex through the PDB (Protein Data Bank) library with the binding partners retrieved from the original oligomer entries. The complex models associated with another chain are deduced from a pre-calculated look-up table, with the best orientation selected by the SPRING-score, which is a combination of threading Z-score, interface contacts, and TM-align match between monomer-to-dimer templates.

These four types of feature datasets together provide a largely comprehensive characterization of isoform pairs. They cover information from sequence, expression, physical interaction as well as amino acid composition. To remove potential uninformative feature datasets, we evaluated each of these 169 datasets against a gold standard, , and removed those datasets with AUC lower than 0.51. Finally, 65 feature datasets were retained for building the final isoform-level network (**Supplementary Table S1**). The predictive values of these feature datasets are very weak with MD values ranging from 0.1 to 0.2. We also randomly partitioned the gold standard into two subsets. The subset containing 20% gold standards were used for screening feature data and the remaining 80% were held out for evaluation of our model. Results were shown **Figure S6**.

***Gene-level gold standard functionally related pairs of the mouse****.* We constructed a gene-level gold standard of functionally related pairs using the Gene Ontology (GO) [^9^](#_ENREF_9), KEGG [^10^](#_ENREF_10), and BioCyc [^11^](#_ENREF_11) databases. Gene Ontology is organized into a hierarchy where broader terms have more genes annotated to each but represent non-specific biological functions, while specific terms have few genes annotated to each. Some of the GO terms are too broad to be experimentally tested, such as ‘metabolic process’, and gene pairs co-annotated to such terms cannot be considered as truly functionally related. We therefore used a list of Gene Ontology terms voted by the biologists, which represent a wide spectrum of experimentally testable biological processes [^12^](#_ENREF_12), and excluded the terms with more than 300 annotated genes. A pair of genes is considered to be functionally related if they are co-annotated to the same specific biological process or involved in the same biological pathway as defined by KEGG or BioCyc. Such a gene pair is defined as positive. The numbers of gold standard gene pairs in GO, KEGG and BioCyc are 641044, 26637 and 11909, respectively. After combining them and removing redundant ones, we obtained 675,124 positive gene pairs in total.

Unlike positive pairs, there is no database that defines two genes as functionally not related. Consistent with previous works in this field , we used random pairs as negatives and fixed a ratio of negatives to positives as 19:1. This ratio serves as the initial prior, and is fixed throughout the iterative process to ensure a consistent prior for Bayesian network modeling.

**Text S2**. **Methods for simulating data**

We simulated a series of scenarios to examine the ability of our algorithm to predict functional relationships at the isoform level. In this simulation study, we set the number of genes to 5000 and as in the real NCBI database, a gene may contain one or several isoforms. The number of positives (functionally related gene pairs) was set to 10, 000. The number of negatives (functionally unrelated gene pairs) was set to 19 times that of positives based on our previous study [^14^](#_ENREF_14). The number of isoform-pair level features was set to 50.

We focused on examining the effects of two factors on the performance of our algorithm. 1, the discriminativeness of the feature data and 2, the fraction of the multi-isoform genes among all genes. For each feature, we simulated that the distributions of the positive examples and the negative examples both follow normal distributions with a standard deviation of 1. Then, the discriminativeness of features is controlled by the Mean Difference (MD) between the population of functionally related isoform pairs and the population of functionally unrelated isoform pairs (**Figure 2**). In our study, three MD values, *i.e.* 0.1, 0.2 and 0.3, were tested. For the second factor, based on the RefSeq, which is a validated database of genes and isoform annotation, a gene may contain a single or multiple isoforms. So, the ratio of multi-isoform genes to the total number of genes (MGR) should be considered. For this ratio, we tested three values: 0.2, 0.3 and 0.5. For example, MGR=0.5 means that half of the genes are multi-isoform genes.

**Text S3. Significant test on real data**

We compared the accuracy of our predictions in terms of area under the precision-recall curve (AUPRC) to the random baseline for significance test. Based on the experimentally identified protein-interaction data (Supplementary Data 3 in ref [^15^](#_ENREF_15)), we obtained a total number of between-gene isoform pairs (two isoforms are from two different genes) is 1304. Each of these 1304 pairs has a prediction score, and 614 out of them are positives (validated) with the remaining being negatives. The AUPRC of our prediction is 0.501. By shuffling the score of all the isoform pairs while keeping their label unchanged, we calculated the AUPRC of the shuffled data which serves as null distribution. Repeating the shuffling 500 times, the null AUPRC, denoted as AUPRC_null_, has a mean = 0.469 with standard deviation=0.012. With the null distribution, we calculated the p-value of our prediction (AUPRC =0.501) using $p=\frac{n(AUPRC<AUPRC_{null})}{N}$, where n(AUPRC<AUPRC_null_) represents the times that AUPRC is observed to be smaller than AUPRC_null_ and N is the total number of shuffling, *i.e.* N=500 in our case. P is calculated to be 0.006, showing that our prediction is significant.

In addition, we also tested our prediction in the whole space of all possible isoform pairs (not only the gene pair in in supplementary data 3 in reference [^15^](#_ENREF_15)). We obtained 2,804 docking pairs calculated by using the protein sequence translated from the DNA sequences of the isoforms provided. Then we randomly generated 2804 isoform pairs and counted the overlap between random and the validated PPI data, giving overlap numbers with 6.6±2.6 as null distribution. The overlap between our data and the validated PPI data is 31, which is significantly higher compared to the null distribution, giving a p-value<1.5×10^-12^.

**Supplementary Table S1. Description of isoform-level genomic data integrated in our work for building mouse isoform networks (SRA: Short Read Archive in NCBI, GEO: Gene Expression Omnibus in NCBI).**

| **Dataset ID** | **Type** | **Database** | **Description** |
| --- | --- | --- | --- |
| SRP012040 | RNA-seq | SRA | GSE36025: Long RNA-seq from ENCODE/Cold Spring Harbor Lab of multiple tissues including ovary, gland, lung, liver etc. |
| SRP006003 | RNA-seq | SRA | GSE27843: Genomic prevalence of heterochromatic H3K9me2 and transcription do not discriminate pluripotent from terminally differentiated cells (RNA-seq) |
| SRP006832 | RNA-seq | SRA | GSE29446: Transcriptome Complexity in Normal and Failing Murine Hearts Revealed by High-Throughput RNA Sequencing |
| SRP007832 | RNA-seq | SRA | GSE31271: Control of Embryonic Stem Cell Lineage Commitment by Core Promoter Factor, TAF3 (RNA-Seq data) |
| SRP002811 | RNA-seq | SRA | GSE22131: High resolution analysis of genomic imprinting in the embryonic and adult mouse brain AND Sex-specific imprinting in the mouse brain |
| SRP010262 | RNA-seq | SRA | GSE35005: Epigenetic dynamics of 5-hydroxymethylcytosine during mouse spermatogenesis. |
| SRP007956 | RNA-seq | SRA | GSE31223: Gene expression of polyoma middle T antigen induced mammary tumors [RNA_Seq : MOLF x PyMT] |
| SRP009464 | RNA-seq | SRA | GSE33979: Novel roles for Klf1 in regulating the erythroid transcriptome revealed by mRNA-seq |
| SRP007827 | RNA-seq | SRA | GSE31385: Chromatin based modeling of transcription rates identifies the contribution of different regulatory layers to steady-state mRNA levels. |
| SRP006165 | RNA-seq | SRA | Sequencing of newly synthesized, preexisting and bulk mRNA |
| SRP007412 | Exon array | GEO | GSE30352: Comparative transcriptome analyses reveal the evolution of gene expression levels in mammalian organs |
| GSE43951 | Exon array | GEO | Conditional knockdown of DNA methyltransferase-1 (Dnmt1) reveals a key role of retinal pigment epithelium integrity in photoreceptor outer segment morphogenesis |
| GSE33009 | Exon array | GEO | Molecular characterization of transport mechanisms at the developing mouse blood-CSF interface: a transcriptome approach |
| GSE40416 | Exon array | GEO | Acute and long-term effects of mutant p53 in vivo [MEF] |
| GSE40415 | Exon array | GEO | Acute and long-term effects of mutant p53 in vivo [B cells] |
| GSE40414 | Exon array | GEO | Acute and long-term effects of mutant p53 in vivo [Thymus] |
| GSE39529 | Exon array | GEO | Foxp3 expression is required for the induction of therapeutic tissue tolerance |
| GSE37246 | Exon array | GEO | SBRI_AB Host response in primary mouse tracheal epitelial cells (mTEC) to influenza infection with PR8, VN or X31 at MOI of 0.015 at 12 hours |
| GSE37052 | Exon array | GEO | Identification of a FOXO3/IRF7 circuit that limits inflammatory sequelae of antiviral responses |
| GSE37051 | Exon array | GEO | Identification of a FOXO3/IRF7 circuit that limits inflammatory sequelae of antiviral responses (expression) |
| GSE40737 | Exon array | GEO | Splicing switch of an epigenetic regulator by RNA helicases promotes tumor-cell invasiveness |
| GSE40527 | Exon array | GEO | Expression Analysis of Whole Thymus and Thymic Tumors on the Sdl Mouse Model |
| GSE40022 | Exon array | GEO | Expression and function of PML-RARA in the multipotent hematopoietic progenitor cells of Ctsg-PML-RARA mice |
| GSE31957 | Exon array | GEO | Effect of miR-144/miR451 expression on TC-1 lung epithelial cell responses to influenza infection for 24 hours |
| GSE31955 | Exon array | GEO | Effect of miR-144/miR451 expression on TC-1 lung epithelial cell responses to influenza infection for 24 hours [Expression] |
| GSE30411 | Exon array | GEO | Clock rescue in the brain restores 12- and 24- hour rhythms in the liver |
| GSE36435 | Exon array | GEO | Expression data from Flt3L-derived bone marrow dendritic cells from WT or Clec9agfp/gfp mice treated or not with dead cells |
| GSE34793 | Exon array | GEO | The General Transcription Factor TAF7 is Essential for Embryonic Development but Not Essential for the Survival or Differentiation of Mature T Cells (MEF data) |
| GSE36348 | Exon array | GEO | The histone demethylase KDM1A sustains the oncogenic potential of MLL-AF9 leukemia stem cells |
| GSE36347 | Exon array | GEO | The histone demethylase KDM1A sustains the oncogenic potential of MLL-AF9 leukemia stem cells (expression data) |
| GSE32359 | Exon array | GEO | Transcriptional study of the response of murine bone marrow-derived macrophages to TLR4 stimulation with LPS |
| GSE32358 | Exon array | GEO | Transcriptional study of oxLDL-induced foam cell formation in WT and Atf3-/- murine bone marrow macrophages |
| GSE36594 | Exon array | GEO | Transcriptome analysis of medulloblastoma tumors in mice |
| GSE36017 | Exon array | GEO | Whole genome sequencing of murine induced Pluripotent Stem (iPS) cell clones |
| GSE35482 | Exon array | GEO | Comparison of gene expression profiles in NOD and Idd9 CD4+ T cells |
| GSE33088 | Exon array | GEO | Developmental time-course of adult cell-type-specific retina genes of amacrine cells |
| GSE33674 | Exon array | GEO | Preservation of cone photoreceptors after a rapid yet transient degeneration and remodeling in cone-only Nrl-/- mouse retina |
| GSE26766 | Exon array | GEO | Interleukin 1b triggers catabolism via a central nervous system-mediated pathway in mice and rat |
| GSE29891 | Exon array | GEO | Systems analysis identifies an essential role for SHARPIN in macrophage TLR2 responses (Affymetrix) |
| GSE29947 | Exon array | GEO | Systems analysis identifies an essential role for SHARPIN in macrophage TLR2 responses |
| GSE29849 | Exon array | GEO | Cell of origin strongly influences genetic selection in a mouse model of T-ALL. |
| GSE23375 | Exon array | GEO | Gene-expression profiles of liver and hepatocellular carcinoma induced by diethylnitrosamine (DEN) in KLF6 +/- and wild type KLF6 mice. |
| GSE24794 | Exon array | GEO | MLL Fusion Proteins Preferentially Regulate a Small Set of Wild Type MLL Target Genes in the Leukemic Genome |
| GSE28889 | Exon array | GEO | Differentially expressed genes and transcript isoforms in mouse NSCs expressing miR-128 |
| GSE26131 | Exon array | GEO | Conserved progression mutations revealed by sequencing a mouse acute promyelocytic leukemia genome |
| GSE26128 | Exon array | GEO | Exon array data from mouse APL tumors |
| GSE26189 | Exon array | GEO | Comparison of exon profiles before and after hormone induction |
| GSE24728 | Exon array | GEO | mCG-PML-RARA alters multipotent hematopoiesis. |
| GSE14387 | Exon array | GEO | RNA degradation in proliferating and differentiated C2C12 muscle precursor cells analyzed on Affymetrix exon arrays |
| GSE23291 | Exon array | GEO | RARA haploinsufficiency modestly influences the phenotype of APL. |
| GSE21757 | Exon array | GEO | Expression data throughout reprogramming of MEF to iPS using a Dox-inducible promoter |
| GSE16967 | Exon array | GEO | Expression data from ERK1/2 null endothelial cells |
| GSE21971 | Exon array | GEO | Alternative splicing is frequent during early embryonic development in mouse |
| GSE20403 | Exon array | GEO | Timecourse of interferon-beta stimulation of mouse bone marrow derived macrophages |
| GSE12766 | Exon array | GEO | Molecular variability of FLT3/ITD mutants and their impact on the differentiation program of 32D cells |
| GSE14534 | Exon array | GEO | Combined genome-wide expression profiling and targeted RNA interference in primary mouse macrophages |
| GSE15998 | Exon array | GEO | Mouse Exon Atlas |
| GSE12185 | Exon array | GEO | HCV tumor promoting effect is dependent on host genetic background |
| GSE12184 | Exon array | GEO | Exon analysis of HCV tumor promoting effect |
| GSE12183 | Exon array | GEO | Transcript analysis of HCV tumor promoting effect |
| GSE13416 | Exon array | GEO | Memory T cell RNA rearrangement by hn RNP LL |
| GSE10599 | Exon array | GEO | SMA mouse tissue exon array analysis |
| GSE11344 | Exon array | GEO | MADS: a New and Improved Method for Analysis of Differential Alternative Splicing by Exon-tiling Microarrays |

**Supplementary Table S2**. GO terms significantly enriched in the local isoform network of NM_001110211.1 and NM_013472.4 of *Anxa6 **.

| Isoform | GO term ID | GO term Name | *p*-value |
| --- | --- | --- | --- |
| NM_001110211.1 | GO:0006944 | cellular membrane fusion | 2.75E-03 |
|  | GO:0061025 | membrane fusion | 3.59E-03 |
|  | GO:0031340 | positive regulation of vesicle fusion | 1.65E-02 |
|  | GO:0007599 | hemostasis | 2.69E-02 |
|  | ***GO:0006906*** | ***vesicle fusion*** | ***9.66E-03*** |
|  | ***GO:0006418*** | ***tRNA aminoacylation for protein translation*** | ***2.11E-02*** |
|  | ***GO:0043039*** | ***tRNA aminoacylation*** | ***2.62E-02*** |
|  | ***GO:0043038*** | ***amino acid activation*** | ***2.62E-02*** |
|  | ***GO:0048284*** | ***organelle fusion*** | ***4.39E-02*** |
|  | ***GO:0031338*** | ***regulation of vesicle fusion*** | ***4.61E-02*** |
| NM_013472.4 | GO:0006944 | cellular membrane fusion | 4.11E-03 |
|  | GO:0061025 | membrane fusion | 5.38E-03 |
|  | GO:0031340 | positive regulation of vesicle fusion | 2.09E-02 |
|  | GO:0007599 | hemostasis | 4.01E-02 |
|  | ***GO:0008360*** | ***regulation of cell shape*** | ***1.35E-02*** |
|  | ***GO:0044699*** | ***single-organism process*** | ***4.20E-02*** |

*, the uniquely enriched GO terms are in **Bold**.

**Reference**

1 Andrews, S., Tsochantaridis, I. & Hofmann, T. Support vector machines for multiple-instance learning. in *Neural Inf. Process Syst. 2003.*

2 Leinonen, R., Sugawara, H. & Shumway, M. The sequence read archive. *Nucleic Acids Res.* **39**, D19-21 (2011).

3 Trapnell, C., Pachter, L. & Salzberg, S. L. TopHat: discovering splice junctions with RNA-Seq. *Bioinformatics* **25**, 1105-1111 (2009).

4 Trapnell, C. *et al.* Differential gene and transcript expression analysis of RNA-seq experiments with TopHat and Cufflinks. *Nat. Protoc.* **7**, 562-578 (2012).

5 David, F. N. The moments of the Z and F distributions. *Biometrika* **36**, 394–403. (1949).

6 Chen, P., Lepikhova, T., Hu, Y., Monni, O. & Hautaniemi, S. Comprehensive exon array data processing method for quantitative analysis of alternative spliced variants. *Nucleic Acids Res.* **39**, e123 (2011).

7 Chou, K.-C. Prediction of protein cellular attributes using pseudo-amino acid composition. *Proteins: Struct., Funct., Bioinf.* **43**, 246-255 (2001).

8 Guerler, A., Govindarajoo, B. & Zhang, Y. Mapping monomeric threading to protein-protein structure prediction. *J. Chem. Inf. Model.* **53**, 717-725 (2013).

9 Ashburner, M. *et al.* Gene Ontology: tool for the unification of biology. *Nat. Genet.* **25**, 25-29 (2000).

10 Kanehisa, M., Goto, S., Kawashima, S., Okuno, Y. & Hattori, M. The KEGG resource for deciphering the genome. *Nucleic Acids Res.* **32**, D277-D280 (2004).

11 Karp, P. D. *et al.* Expansion of the BioCyc collection of pathway/genome databases to 160 genomes. *Nucleic Acids Res.* **33**, 6083-6089 (2005).

12 Myers, C. L., Barrett, D. R., Hibbs, M. A., Huttenhower, C. & Troyanskaya, O. G. Finding function: evaluation methods for functional genomic data. *BMC Genomics* **7**, 187 (2006).

13 Troyanskaya, O. G., Dolinski, K., Owen, A. B., Altman, R. B. & Botstein, D. A Bayesian framework for combining heterogeneous data source for gene function prediction (in Saccharomyces cerevisiae). *Proc. Natl Acad. Sci. USA* **100**, 8348-8353 (2003).

14 Guan, Y. *et al.* Tissue-specific functional networks for prioritizing phenotypes and disease genes. *PLOS Comput. Biol.* **8**, e1002694 (2012).

15 Corominas, R. *et al.* Protein interaction network of alternatively spliced isoforms from brain links genetic risk factors for autism. *Nat. Commun.* **5**, 3650 (2014).
